# Plasma MicroRNA Panel Predicts Early Tumor Recurrence in Patients with Hepatocellular Carcinoma after Liver Transplantation

**DOI:** 10.7150/jca.59612

**Published:** 2021-10-22

**Authors:** Ao Huang, De-Zhen Guo, Yu-Peng Wang, Guo-Huan Yang, Qi-Man Sun, Xin Zhang, Yi-Feng He, Kang Song, Xiao-Wu Huang, Xin-Rong Yang, Jia Fan, Jian Zhou, Jie Hu

**Affiliations:** 1 Department of Liver Surgery and Transplantation, Liver Cancer Institute, Zhongshan Hospital, Fudan University; Key Laboratory of Carcinogenesis and Cancer Invasion (Fudan University), Ministry of Education; Shanghai Key Laboratory of Organ Transplantation, Zhongshan Hospital, Fudan University, Shanghai, 200032, China.; 2 Institute of Biomedical Sciences, Fudan University, Shanghai, 200032, China.; 3 State Key Laboratory of Genetic Engineering, Fudan University, Shanghai, 200032, China.

**Keywords:** hepatocellular carcinoma, liver transplantation, early recurrence, liquid biopsy, microRNA.

## Abstract

**Background:** This study aimed to evaluate the role of plasma microRNA panel (miR-122, miR-192, miR-21, miR-223, miR-26a, miR-27a and miR-801) for prediction and surveillance of early tumor recurrence in hepatocellular carcinoma (HCC) patients who had undergone liver transplantation (LT).

**Methods:** The expression of plasma microRNA panel was assayed in 193 HCC patients (training cohort, n =151; validation cohort, n = 42). Sensitivity and specificity for detecting post-transplant HCC recurrence, and the relationship of microRNA panel expression with clinical characteristics were analyzed accordingly. The prognostic value of microRNA panel was compared with that of AFP (alpha-fetoprotein) and DCP (Des-gamma-carboxyprothrombin). Cox regression analyses were used to evaluate independent prognostic factors.

**Results:** In the training cohort, the rate of positive plasma microRNA panel status at 7-14 days after LT (late phase; 44.2%) decreased than that before (76.2%, *P* < 0.001) and 1-6 days after LT (early phase; 78.5%, *P* < 0.001). At late phase, positive microRNA panel status correlated with higher early tumor recurrence rate (one year after LT) than negative status (45.9% vs 10.7%; *P* < 0.001). Patients with persistent positive microRNA panel status both before and after LT had the highest early tumor recurrence rate in this cohort (54.9%, *P* < 0.001). The results were consistent in the validation cohort. Cox regression analysis found that positive plasma microRNA panel status at late phase was the only independent risk factor for early recurrence (HR: 4.903, 95% CI = 2.195 - 10.951; *P* < 0.001). Dynamic monitoring demonstrated plasma microRNA panel status changed from negative to positive earlier than AFP and DCP upon recurrence, and the median time between positivity of plasma microRNA and imaging evidence of recurrence was 2.4 (0.5-10.0) months.

**Conclusions:** Plasma microRNA panel could be a noninvasive biomarker for prediction and surveillance of early tumor recurrence in HCC patients who have undergone LT.

## Introduction

Hepatocellular carcinoma (HCC) is the fourth most common cause of cancer-related death worldwide, and China has approximately half of the global incidence and mortality of HCC 1. The treatment choice of HCC depends on tumor burden, liver function and physical status. As a curative method, liver transplantation (LT) could treat both the tumor and the underlying liver disease, achieving much more satisfactory outcome than other modalities 2. However, even with strict patient selection criteria and careful postoperative management, tumor recurrence still occurs with a probability of 8-20% in a median of 13 months (range 2-132 months) after LT 3. Although the risk of HCC recurrence drastically increases when the tumor features exceed Milan criteria, more LTs have been done on HCCs beyond Milan criteria nowadays and this has made post-LT tumor recurrence more noteworthy 4.

The reason for tumor recurrences after LT largely lies in the growth of occult metastases in extrahepatic organs and the colonization of circulating HCC cells in graft after transplantation. Early recurrence (commonly within a year of LT) stands as an important limitation for long-term survival of HCC patients after LT 5. Generally, blood based biomarker test is ideal for dynamic surveillance but the role of AFP (alpha-fetoprotein) is less satisfactory 6 and other modalities including CT scan, bone scintigraphy could not be done dynamically and are less sensitive. Although prognostic scoring systems have been developed for recurrence risk prediction and stratification of patients, successful translation of this information into tumor surveillance in clinical practice has yet to be demonstrated 7. Therefore, novel biomarkers or strategies for predicting early recurrence following LT are still needed.

The era of liquid biopsy has brought new hope for clinical management of HCC 8 and it's thus reasonable to propose liquid biopsy based method to predict early recurrence after LT. Previously, we had discovered and validated a plasma microRNA panel (miR-122, miR-192, miR-21, miR-223, miR-26a, miR-27a and miR-801) with a high diagnostic accuracy of HCC and this panel could also differentiate HCC from healthy control, patients with chronic hepatitis B , and liver cirrhosis respectively 9. Currently, this microRNA panel has been translated into commercial test kit, certified by National Medical Product Administration, and applied in China as companion diagnostic and monitoring test for HCC. Noticeably, we have also used this plasma microRNA panel kit in the management of HCC patients who received LT. Herein, we reported for the first time that the performance of this plasma microRNA panel in prediction and surveillance of early tumor recurrence in HCC patients who had undergone LT.

## Materials and Methods

### Patient enrollment and LT

**Figure 1:**
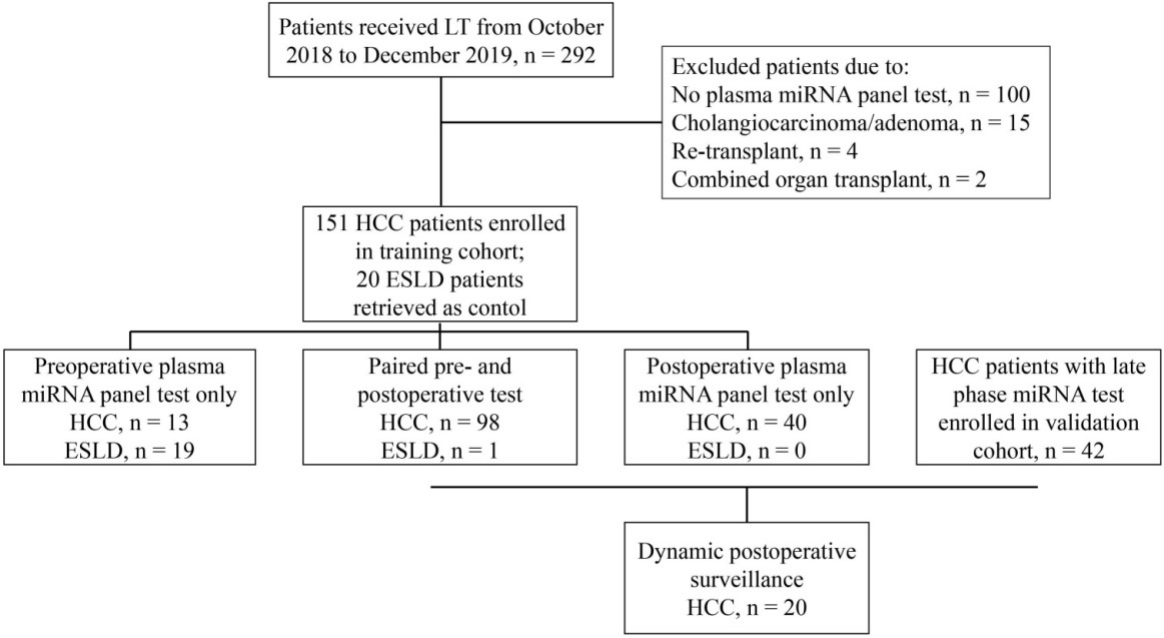
Flow chart for patient selection strategy in this study.

Between October 2018 and December 2019, HCC patients treated at Zhongshan Hospital, Fudan University were retrospectively reviewed and enrolled in the training set (Figure 1) under the following criteria: (1) diagnosed with and pathologically confirmed HCC without simultaneous or previous history of malignancy in other organs; (2) successfully underwent orthotopic liver transplantation; (3) received at least one plasma microRNA panel test. ESLD (end-stage liver disease) patients who underwent LT and had microRNA panel test during the same period were retrieved as control. HCC patients who were treated from January to June 2020 and had late phase miRNA tests were enrolled in the validation set using the same criteria (Figure 1). All liver grafts were from donation after circulatory death, allocated by the China Organ Transplant Response System and procured, preserved and transported by Organ Procurement Organizations. No organs were acquired from executed prisoners. For patients with HCC, candidates for LT were selected based on the Shanghai Fudan Criteria 10. When patients with tumors exceeded the above criteria, salvage LTs were still performed if no extrahepatic metastasis existed and under the premise that the patients knew the risk and benefit and gave informed consent. After LT, patients were routinely given immunosuppresants and in patients with high probability of tumor recurrence, adjuvant treatment including targeted drugs and/or systemic chemotherapy was initiated one month after discharge. This retrospective study has been censored and approved by the Ethics Committee of Zhongshan Hospital, Fudan University (Approval Number: B2020-402).

### Plasma microRNA panel test and results interpretation

The plasma microRNA panel was tested before LT and repeated once or several times after LT. Within four hours of venipuncture, blood samples were first processed by a two-step centrifugation method: first spun at 1,300 g for 20 minutes to remove the majority of blood cells and a second spin at 14,000 g for another 10 minutes to remove the cellular debris. The plasma microRNA was diluted with preservative fluid (JUSBIO SCIENCES, Shanghai, FD05059) and tested with plasma microRNA testing kit (JUSBIO SCIENCES, Shanghai, HCC9655) according to the manufacturer's protocol. MiR-1228 was used as endogenous control as previously reported 11. The Ct value of each miRNA was calculated and dCt = Ct miRNA- Ct miR-1228. The formula for calculating the value of microRNA panel was: -1.9449 + 0.10633 × dCt miR-21 + 0.10219 × dCt miR-26a - 0.012441 × dCt miR-27a - 0.28902 × dCt miR-122 - 0.32779 × dCt miR-192 + 0.25855 × dCt miR-223 - 0.029515 × dCt miR-801. The value < -0.5 was considered to be negative plasma microRNA panel status while ≥ -0.5 was positive status.

### Data collection and follow-up

Baseline clinicopathological features including epidemiology, etiology, treatment history, laboratory test results, pathological diagnosis, tumor number, tumor size, tumor differentiation, liver cirrhosis, lymph node metastasis, microvascular invasion (MVI) were recorded. Generally, patients were examined bi-weekly or monthly during the first three months after LT and every 2-3 months afterwards. During each follow-up, liver function, AFP, DCP (Des-gamma-carboxyprothrombin) and abdominal ultrasonography were tested. Enhanced magnetic resonance imaging was applied when suspicious signs of recurrence were observed, such as increased AFP, DCP, and abnormal ultrasonography imaging. Positron emission tomography/computed tomography was used to evaluate extrahepatic metastasis when necessary. Tumor recurrence or extrahepatic metastasis was confirmed by imaging tools and in some patients with resectable recurrences, by pathological diagnosis. The follow-up of patients continues until December 2020.

### Statistical analysis

All statistical analyses were performed with R software version 3.6.3. All categorical variables were presented as percentages and compared between groups using Chi-square test or Fisher's exact test. All tests were two-tailed and P value less than 0.05 was considered statistically significant. Kaplan-Meier Survival analysis was applied to measure TTR (time to recurrence) in patients. To identify the influence of plasma microRNA panel and other clinicopathological factors on TTR, univariate analysis was performed using Cox regression analysis and variables with statistical significance (*P* < 0.05) were selected as candidate variables for multivariate analysis. Hazard ratio (HR) and 95% confidence interval (95% CI) were calculated accordingly. The overall predictive performance was measured by area under curve (AUC) of the receiver operating characteristic (ROC) curve, with a value of 0.5 and 1.0 indicating no and perfect predictive ability, respectively.

## Results

### Clinicopathological features

During the study period, 151 HCC patients who had received LT and got at least one plasma microRNA test were enrolled in the training set (Figure 1, Table 1). Most HCCs were HBV associated (131, 86.8%), the median tumor number was three and the median tumor size was 3.5 cm. Of all HCC patients, 46 (30.5%), 60 (39.7%), and 67 (44.4%) fulfilled the Milan, UCSF and Shanghai Fudan criteria respectively. Prior to LT, 82 (54.3%) patients had received anti-tumor treatment, including hepatictomy, transcatheter arterial chemoembolization (TACE), or targeted therapy. Twenty ESLD patients who also received LT and got at least one plasma microRNA test at the same period were also retrieved as control (Figure 1, Table 1). As to ESLD patients, liver cirrhosis from HBV infection was the main etiology (12, 60%), followed by alcoholic (3, 15%), schistosomiasis (1, 5%) and idiopathic cirrhosis (4, 20%). The follow-up ranged from 11.2 to 25.1 months with the median time of 16.4 months. Of all patients, no 30-day mortality happened and 2 patients died from complications within 90 days after LT (90-day mortality, 1.17%). During the follow-up, 42 HCC patients had tumor relapses (recurrence rate, 27.8%): 7 had intrahepatic recurrence only, 27 presented with extrahepatic metastasis, and other 8 patients suffered both intrahepatic recurrence and extrahepatic metastasis.

### Diagnostic accuracy of plasma microRNA panel for HCC in real-world setting

**Figure 2:**
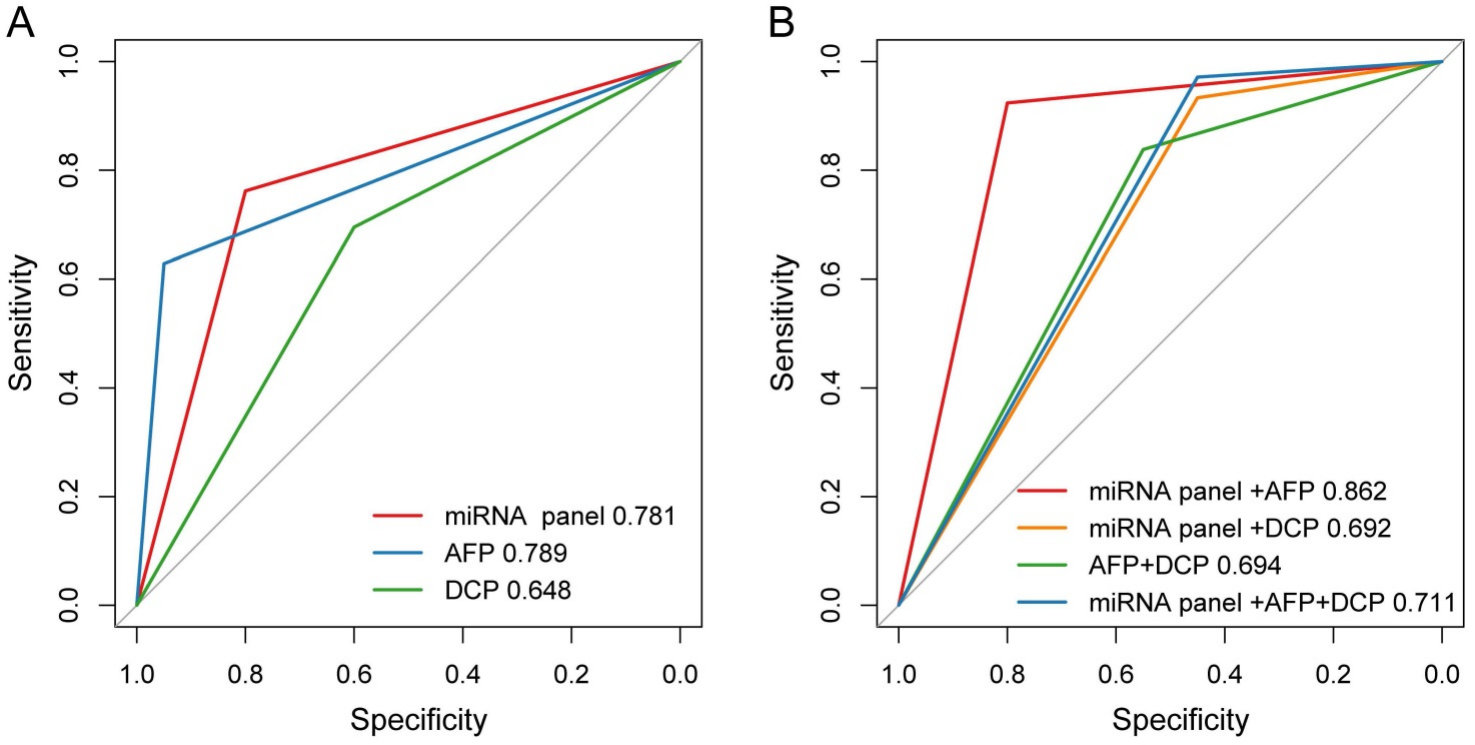
The diagnostic performance of AFP, DCP and plasma microRNA panel for HCC. (A) The AUCs of AFP, DCP and plasma microRNA panel in ROC curve. (B) The AUCs of different combinations of AFP, DCP and plasma microRNA panel in ROC curve.

Among the 151 HCC patients included in present study, 105 patients did miRNA tests before LT and the positive rate was 76.2% (80/105). Meanwhile, these patients were also tested for AFP and DCP, of which the positive rates were 62.9% (66/105) and 69.5% (73/105) respectively. Of all the patients, the sensitivity and specificity of plasma microRNA panel for detecting HCC were 76.2% and 80.0%, 62.9% and 95.0% for AFP, and 69.5% and 60.0% for DCP, respectively. The plasma microRNA panel had AUC comparable with AFP and higher than DCP for diagnosing HCC (Figure 2A). Specially, in AFP negative patients (39 HCC and 19 ESLD cases), the sensitivity and specificity of plasma microRNA panel was 79.5% and 84.2%.

We also evaluated the performance of different combinations of these three biomarkers for HCC diagnosis. The positivity was defined as any marker being positive in the combination and negativity was defined as all markers being negative in the combination. We found that the combination of microRNA panel with AFP demonstrated better diagnostic performance for distinguishing HCC from ESLD, with a sensitivity of 92.4% and a specificity of 80.0%. The diagnostic performance of microRNA panel with AFP (AUC, 0.862) was higher than those of the combinations of microRNA panel with DCP (AUC, 0.692; *P* = 0.003), AFP with DCP (AUC, 0.694; *P* = 0.024), or the combination of all three biomarkers (AUC, 0.711; *P* = 0.007, Figure 2B).

### Liver function affects plasma microRNA status after LT

**Figure 3:**
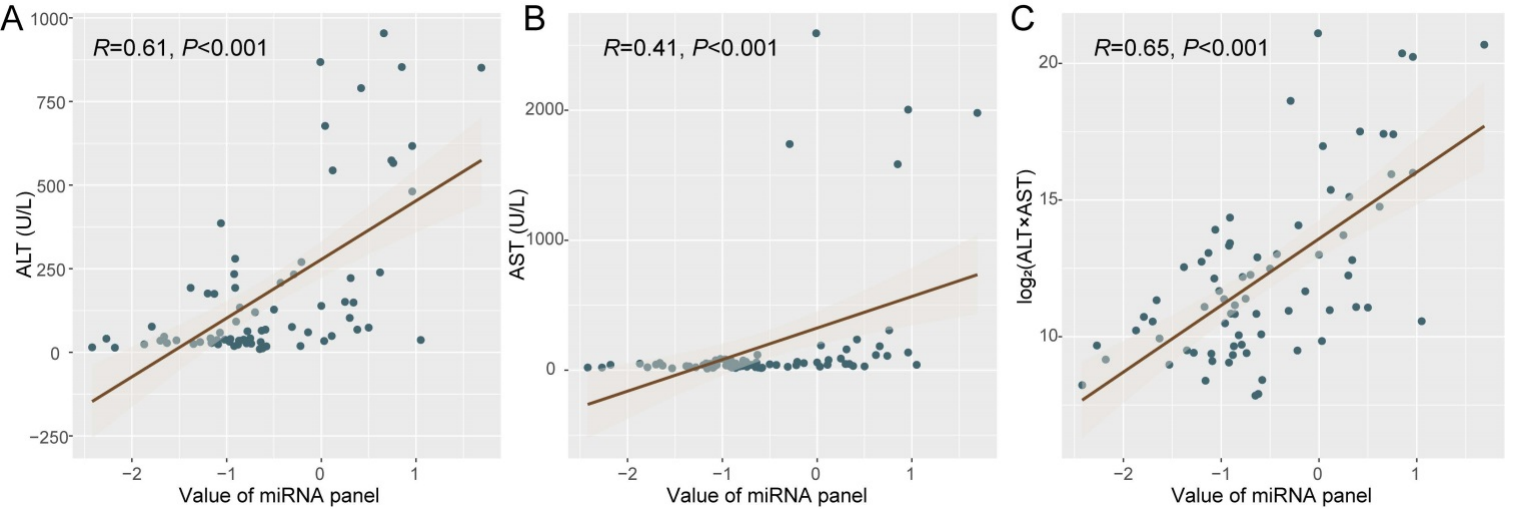
The correlation between liver function and plasma microRNA panel status. Correlation analysis was made between plasma microRNA panel status with ALT (A), AST (B), and Log_2_(ALT*AST) (C) respectively.

Since the microRNAs consisting these panels derive from not only tumor but also normal tissues, the ischemia-reperfusion and surgical injury during LT may thus affect the microRNA release by liver cells of the graft and change plasma miRNA status. We then tried to analyze the dynamic change of plasma microRNA panel during perioperative period and its potential association with liver function. Specially, we focused on patients with negative miRNA status before LT (n = 24). Interestingly, it was identified that the value of plasma microRNA panel was associated with ALT (alanine aminotransferase, R = 0.61, *P* < 0.001, Figure 3A) and AST (aspartate aminotransferase, R = 0.41, *P* < 0.001, Figure 3B), and specifically with log_2_(ALT*AST) (R = 0.65, *P* < 0.001, Figure 3C). Other indexes such as TB (total bilirubin, R = 0.10, *P* = 0.40), ALB (albumin, R = 0.03, *P* =0.83), and GGT (γ-glutamyl transpeptidase, R = 0.04, *P* = 0.71) were not associated with the value of plasma microRNA panel.

### Association of plasma microRNA status with early recurrence after LT

We first compared the preoperative and postoperative miRNA positive status rates. Considering some patients received multiple microRNA tests after LT, we defined postoperative miRNA positivity as at least one positive miRNA status in the multiple tests before discharge and no differences were found between preoperative (n = 105) and postoperative (n = 138) miRNA positive status rates (76.2% vs 73.2%, *P* = 0.595). To identify the association between plasma microRNA status and early recurrence after LT, we compared the tumor recurrence rates between patients with different preoperative or postoperative plasma microRNA status, respectively. The tumor recurrence rates were comparable between patients with negative and positive miRNA status, both preoperatively (28.0% vs 31.2%, *P* = 0.758) and postoperatively (18.9% vs 30.7%, *P* = 0.170).

**Figure 4:**
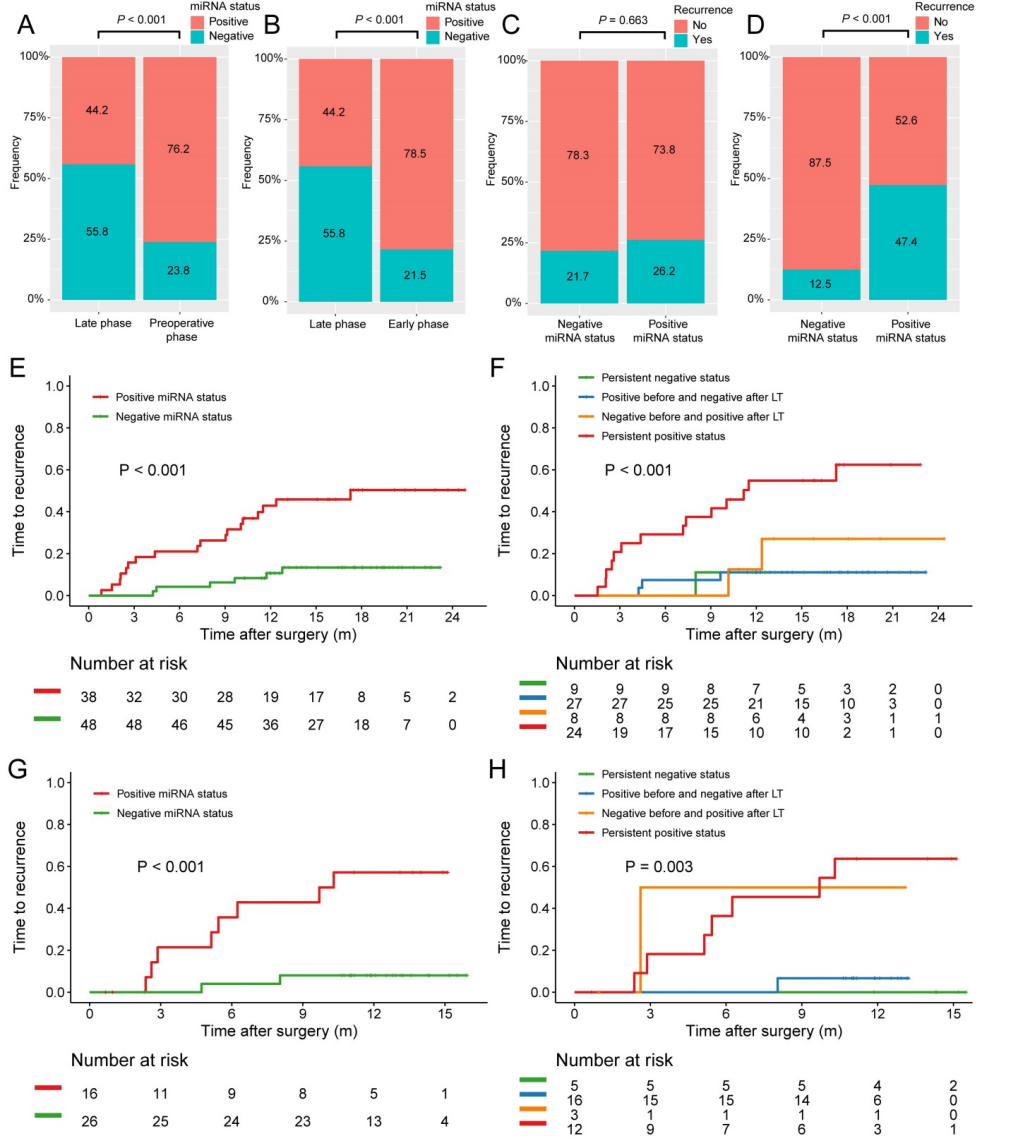
Proportion of plasma microRNA panel status at different stages and its association with early tumor recurrence after LT. (A) The proportion of patients with positive postoperative plasma microRNA panel status at late phase decreased when compared with that of preoperative status. (B) The proportion of patients with positive postoperative plasma microRNA panel status at late phase decreased when compared with that of early phase. (C) The tumor recurrence rates between patients with different microRNA status at early phase after LT. (D) The tumor recurrence rates between patients with different microRNA status at late phase after LT. (E) Kaplan-Meier estimate of 1-year TTR in patients of the training cohort with different late phase plasma microRNA panel status. (F) Kaplan-Meier estimate of 1-year TTR in four groups of patients of the training cohort with paired plasma microRNA panel status. (G) Kaplan-Meier estimate of 1-year TTR in patients of the validation cohort with different late phase plasma microRNA panel status. (H) Kaplan-Meier estimate of 1-year TTR in four groups of patients of the validation cohort with paired plasma microRNA panel status.

Since abnormal liver function correlated with positive microRNA status and it's quite common to observe increased ALT and AST levels within 7 days after LT, we then classified the postoperative microRNA test results into two groups based on the testing date: the early phase group (n = 107) got tested within 6 days after LT whereas the late phase group (n = 86) had the assay within 7-14 days after LT irrespective of the early phase results, if applicable. Indeed, during the early phase after LT, patients tend to had poorer liver function (median ALT, 357.0 U/L vs 83.0 U/L, *P* < 0.001; median AST, 138.0 U/L vs 31.0 U/L, *P* < 0.001). The positive miRNA status rate at late phase (44.2%) significantly decreased when compared with that of preoperative phase (76.2%, *P* < 0.001, Figure 4A) or early phase (78.5%, *P* < 0.001, Figure 4B). In the early group, positive microRNA status did not correlate with recurrence (26.2% vs 21.7%, *P* = 0.663, Figure 4C) while in the late group, patients with positive microRNA status had significantly higher early tumor recurrence rate (47.4% vs 12.5%, *P* < 0.001, Figure 4D).

### Positive miRNA status at late phase after LT independently predicted early tumor recurrence

Kaplan-Meier survival estimate was then employed and no associations between preoperative, postoperative, or early phase plasma microRNA positive status with TTR were found. Only in the late group, patients with positive microRNA status was found to have significant poorer TTR after LT when compared with patients with negative ones (1-year TTR, 45.9% vs 10.7%; *P* < 0.001; Figure 4E). Based on the microRNA status change before and after LT, the patients were further stratified into four groups: persistent positive status, persistent negative status, positive before and negative after LT, and negative before and positive after LT. Interestingly, patients with persistent positive status had the highest recurrence rate while the other three groups had similar rates (1-year TTR, 54.9% vs 11.1% vs 11.1% vs 27.1%; *P* < 0.001; Figure 4F).

The prognostic value of miRNA status at late phase was further validated in the validation cohort consisting of 42 HCC patients who all had at least one late phase miRNA test (Figure 1). The baseline characteristics of HCC patients with late phase miRNA status in the training and validation cohorts were comparable (Table 1). The TTR for patients with positive microRNA status at late phase was significantly shorter those with negative ones (1-year TTR, 57.1% vs 8.0%; *P* < 0.001; Figure 4G). Stratified by the microRNA status change before and after LT, persistent positive status also showed the highest recurrence rate (1-year TTR, 63.6% vs 0.0% vs 6.7% vs 50%; *P* = 0.003; Figure 4H).

Cox proportional hazards regression model was further employed for the whole cohort to figure out whether plasma microRNA status at late phase was an independent risk factor of early tumor recurrence after LT. Indeed, both univariate and multivariate cox regression analysis found plasma microRNA status in the late phase was the only independent risk factor of early recurrence postoperatively (HR = 4.903, 95% CI = 2.195-10.951, *P* < 0.001; Table 2).

### Surveillance of tumor recurrence by dynamic plasma microRNA testing

**Figure 5:**
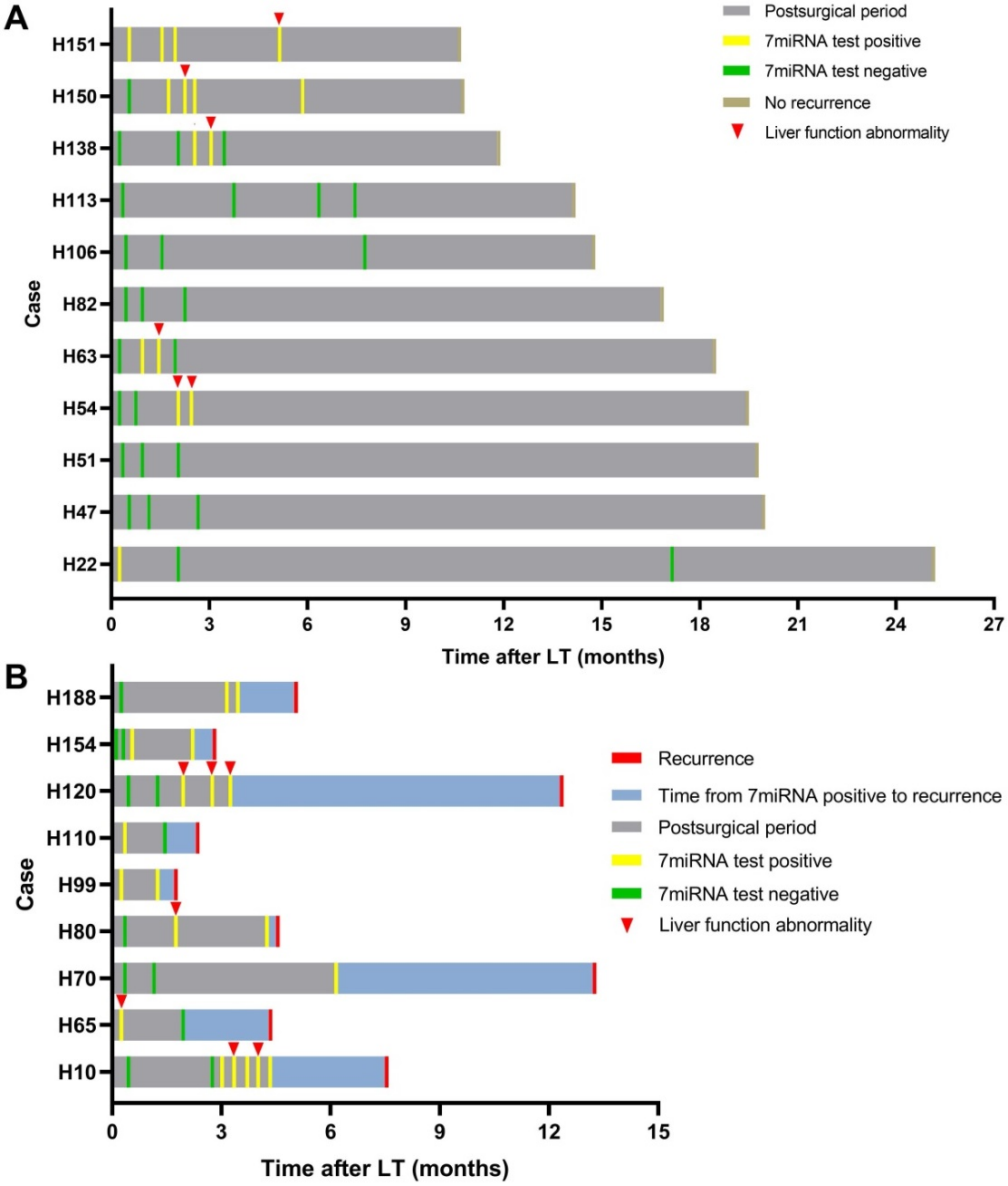
Serial detection results of plasma microRNA panel after LT. Summaries of clinical events for patients with plasma microRNA tests after LT without (A) and with recurrence (B).

We further tested whether the plasma microRNA test could predict tumor recurrence in dynamic surveillance manner. Totally, 20 HCC patients had undergone multiple microRNA tests and 9 patients eventually had tumor recurrence. Consistent with abovementioned results, the plasma microRNA in the early phase was positive in most patients whereas in the late phase, it went negative in patients without recurrence but continued to be positive in recurrent cases (Figure 5). Although microRNA status occasionally went positive in patients without recurrence during follow-up, such changes largely coincided with liver function abnormality: patients H54, H63, and H138 had transplant rejections and patients H150 and H151 experienced idiopathic liver injury. The microRNA went negative in these patients after rejection or abnormal liver function was cured. The other 7 patients (H22, H47, H51, H82, H106, H113) had persistent negative plasma microRNA status without presenting abnormal liver function.

**Figure 6:**
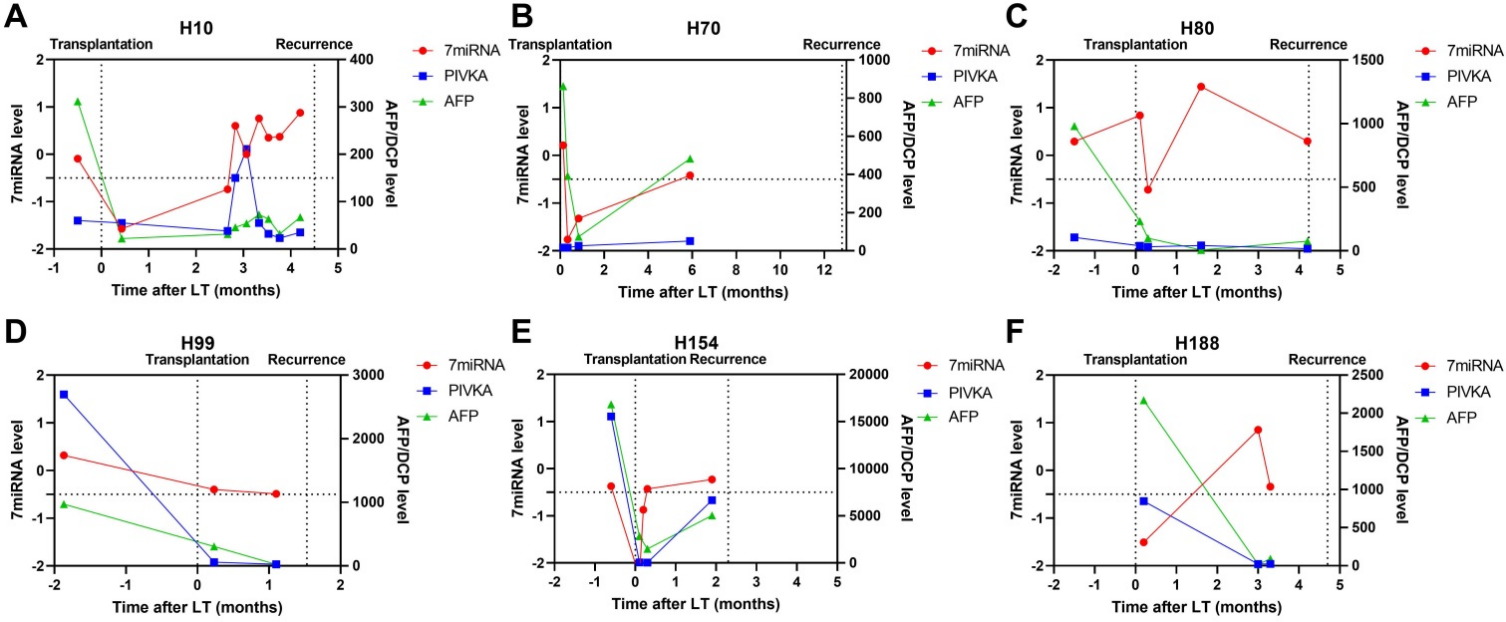
Case-by-case study of plasma microRNA panel status for tumor recurrence surveillance in comparison with AFP and DCP. The Schematic diagram summarizing clinical events and results of blood tests for patient H10 (A), H70 (B), H80 (C), H99 (D), H154 (E), and H188 (F).

In patients with tumor recurrence, we also observed abnormal liver function resulted positive plasma microRNA status (Figure 5; patient H10) but such status continued even after liver injury had been treated. Patient H80 and H120 had both developed transplant rejections and the microRNA status remained positive until recurrence. Besides, we observed plasma microRNA status changed positive ahead of AFP and DCP increase or imaging examinations upon tumor recurrence. For example, in patient H10, the microRNA status kept positive while AFP and DCP fluctuated around the cut-off value (Figure 6). The median time between postoperative plasma microRNA first changed positive to tumor recurrence was 2.4 (0.5-10.0) months.

## Discussions

Liver transplantation may probably be the most effective treatment strategy for HCC since it removes not only the tumor but also the diseased liver remnant. However, tumor recurrence after transplant could still happen, especially in patients who have tumors beyond the Shanghai Fudan criteria. In China, more HCCs are diagnosed at advanced stage and some patients receive salvage LT; even in western world, the post-Milan-criteria era has witnessed the growing experience with downstage of tumor via locoregional therapies to become a candidate for transplantation 12, 13. These patients are generally at relatively high risk of tumor recurrence after LT. Despite enormous work has been done to better stratify LT candidates with recurrence following transplant, currently there is no evidence to support specific post-LT HCC recurrence surveillance and treatment strategies 14. Thus, the introduce of novel biomarkers or methods to predict and monitor HCC recurrence after LT is of significant importance for appropriate allocation of the limited donor liver organs and to prolong the survival of these patients.

Previous studies have found miRNAs hold the potential as biomarkers for early detection of HCC 15, liver transplantation 16 and identified recurrence-related miRNA profiles in HCC following liver transplantation 17-19; however, those miRNA profiles were recipient liver tumor tissue derived and had not been validated in blood-based assays, making it inappropriate for tumor surveillance. Liquid biopsy has been widely used in the management of cancers including HCC, especially in prediction, prognosis and dynamic monitoring 8. Compared with traditional tumor biomarkers (such as AFP and DCP in HCC), the analytes assayed as liquid biopsy bear high sensitivity, specificity, and could be detected in dynamical manner, enabling consecutive tumor progression warning. Circulating cell-free miRNA is one of such analytes used in liquid biopsy. Application of circulating plasma/serum miRNA in the early detection, diagnosis, and prognosis of HCC has been frequently reported whereas few studies had explored circulating plasma miRNA in HCC patients undergone LT.

Herein, the plasma miRNA panel showed predictive value for tumor recurrence in patients treated by LT. Generally, early HCC recurrence happens within the first year of LT and portends the worst prognosis. This could be the consequence of circulating HCC cells or clusters engrafting and growing in the graft organ after LT. Such residual disease could be hardly found by image or predicted by conventional biomarkers. Tumor specific markers are the potential solution. Indeed, recent study from our team has found circulating tumor cells could evaluate the recurrence risk following LT in patients with HCC 20.

To our knowledge, this is the first time that circulating plasma miRNA panel has been used in the prediction and surveillance of tumor recurrence of HCC following LT. Although previous studies had demonstrated that preoperative miRNAs either from recipients' tumor tissues or serum samples could predict HCC recurrence after LT 17, 21, 22, such result were less likely to be translated into clinical practice since tissue sample had the inherent limitations of intratumoral heterogeneity and unsuitableness for dynamical follow-up and serum was intrinsically inferior to plasma for liquid biopsy. Being different from former studies, the miRNA panel used herein was enriched from plasma and not newly discovered or unverified: our former research found the plasma miRNA panel could effectively differentiate HCC from healthy, chronic hepatitis B, and liver cirrhosis respectively.

In this study, we also observed the association between abnormal liver function and positive microRNA status. During the early phase after LT, ischemia-reperfusion related hepatic injury and surgery related internal environment homeostasis imbalance result in hepatic cellular damage and inflammatory cascade. Since the miRNAs in this panel derives not only from tumor cells but also normal tissues, exceptional miRNA release upon hepacytes injury or pathophysiological conditions may thus lead to status change. The fact that miRNA status changed to positive upon graft rejection and went negative after immunosuppression treatment confirmed abovementioned viewpoint. Indeed, circulating miRNA has been reported as an early noninvasive diagnostic biomarker of allograft rejection and transplant failure 23, 24. Ng et al reported that the level of circulating miR-1246 at 2-hour after portal vein reperfusion positively correlated with serum AST and ALT levels in HCC recipients after LT and *in vitro* experiments found the injury-induced activation and differentiation of macrophages significantly elevated the expression and secretion of miR-1246 25. miR-122, one component in this miRNA panel, was a sensitive biomarker which elevated in LT patients with liver injury and graft rejection, and positively correlated with aminotransferase levels 26, 27. Another component, miR-146a also correlated with cellular rejection 27. Thus, plasma miRNA panel status within the early phase after LT might largely reflect cellular injury rather than residual disease.

There existed several issues to be solved before the wide application of this plasma microRNA panel in clinical practice. First, this is a retrospective single-center based study with unavoidable selection and information biases. Second, most patients enrolled in this study had the background of HBV-related cirrhosis and the role of this microRNA panel needed to be validated in HCC patients with other etiologies, including hepatitis C virus infection, alcohol abuse and nonalcoholic steatohepatitis. Third, the role of abnormal liver function and graft rejection in the result interpretation of this plasma microRNA panel should be clearly clarified. Furthermore, despite HCC patients treated with LT, HCC patients who receive hepatectomy would be more suitable for this test since tumor recurrence, especially early tumor recurrence within 2 years after surgery (most likely the consequence of intrahepatic occult metastasis from primary HCC), stands as the main obstacle for long-term survival. If patients with occult metastasis could be timely predicted postoperatively, adjuvant treatments such as TACE and targeted therapy might be precisely prescribed and delay or even eradicate the development of recurrence. Thus, external validation cohort with prospective and multi-center design is needed.

This study demonstrated that plasma miRNA panel was an effective biomarker for prediction of early HCC recurrence after liver transplantation. Although abnormal liver function resulted positive plasma miRNA panel status may complicate the situation, such change could also herald hepatic injury and serial detection could reduce the influence as well as enable dynamic surveillance, which provided additional chances for earlier therapeutic intervention.

## Figures and Tables

**Table 1:** Demographic and clinicopathologic features of retrieved patients

Variables	HCC patients (n = 151)	ESLD Patients (n = 20)	Patients with miRNA tests at late phase after LT		
			Training (n = 86)	Validation (n = 42)	*P*
Gender					0.806
Female	17 (11.3)	7 (35.0)	77 (89.5)	37 (88.1)	
Male	134 (88.7)	13 (65.0)	9 (10.5)	5 (11.9)	
Age (y)					0.451
≤ 50	45 (29.8)	7 (35.0)	27 (31.4)	16 (38.1)	
>50	106 (70.2)	13 (65.0)	59 (68.6)	26 (61.9)	
HBsAg					0.143
Negative	20 (13.2)	8 (40.0)	10 (11.6)	9 (21.4)	
Positive	131 (86.8)	12 (60.0)	76 (88.4)	33 (78.6)	
Cirrhosis					0.716
No	33 (21.9)	0 (0.0)	20 (23.3)	11 (26.2)	
Yes	118 (78.1)	20 (100.0)	66 (76.7)	31 (73.8)	
Child-Pugh					0.258
A	103 (68.2)	6 (30.0)	64 (74.4)	35 (83.3)	
B-C	48 (31.8)	14 (70.0)	22 (25.6)	7 (16.7)	
AFP (ng/mL)					0.057
≤ 20	75 (49.7)	19 (95.0)	44 (51.2)	14 (33.3)	
>20	76 (50.3)	1 (5.0)	42 (48.8)	28 (66.7)	
DCP					0.378
≤ 40	47 (31.1)	12 (60.0)	28 (32.6)	17 (40.5)	
>40	104 (68.9)	8 (40.0)	58 (67.4)	25 (59.5)	
Tumor size					0.152
≤ 5 cm	110 (72.8)		62 (72.1)	25 (59.5)	
>5 cm	41 (27.2)		24 (27.9)	17 (40.5)	
Tumor number					0.172
Single	36 (23.8)		19 (22.1)	14 (33.3)	
Multiple	115 (76.2)		67 (77.9)	28 (66.7)	
Edmondson stage					0.319
I-II	63 (41.7)		35 (40.7)	21 (50.0)	
III-IV	88 (58.3)		51 (59.3)	21 (50.0)	
MVI					0.317
No	56 (37.1)		31 (36.0)	19 (45.2)	
Yes	95 (62.9)		55 (64.0)	23 (54.8)	
CNLC stage					0.256
I-IIa	73 (48.3)		42 (48.8)	25 (59.5)	
IIb-IV	78 (51.7)		44 (51.2)	17 (40.5)	
BCLC stage					0.243
A	45 (29.8)		24 (27.9)	16 (38.1)	
B-C	106 (70.2)		62 (72.1)	26 (61.9)	
Milan criteria					0.304
Yes	46 (30.5)		25 (29.1)	16 (38.1)	
No	105 (69.5)		61 (70.9)	26 (61.9)	
UCSF criteria					0.317
Yes	60 (39.7)		31 (36.0)	19 (45.2)	
No	91 (60.3)		55 (64.0)	23 (54.8)	
Fudan criteria					0.717
Yes	67 (44.4)		36 (41.9)	19 (45.2)	
No	84 (55.6)		50 (58.1)	23 (54.8)	

**Table 2:** Univariate and multivariate Cox regression analyses for time to recurrence in all HCC patients

Variables	Univariate analysis		Multivariate analysis	
	HR (95% CI)	P value	HR (95% CI)	P value
Gender (female vs. male)	1.071 (0.377-3.043)	0.897		
Age (y) (>50 vs.≤50)	0.871 (0.431-1.761)	0.701		
HBsAg (positive vs. negative)	1.391 (0.489-3.955)	0.536		
Liver cirrhosis (Yes vs. No)	0.607 (0.295-1.248)	0.175		
Child-Pugh class (A vs. B-C)	0.855 (0.372-1.964)	0.712		
AFP (ng/mL) (>20 vs. ≤ 20)	1.628 (0.804-3.295)	0.176		
Tumor size (cm) (>5 vs. ≤ 5)	3.088 (1.570-6.074)	0.001	1.753 (0.820-3.748)	0.148
Tumor number (multiple vs. single)	1.410 (0.614-3.238)	0.419		
Edmondson stage (Ⅲ-Ⅳ vs. Ⅰ-Ⅱ)	2.788 (1.261-6.163)	0.011	1.691 (0.731-3.914)	0.220
Microvascular invasion (Yes vs. No)	2.815 (1.225-6.470)	0.015	1.702 (0.682-4.248)	0.255
Plasma miRNA status (positive vs negative)	5.896 (2.666-13.039)	<0.001	4.903 (2.195-10.951)	<0.001

## References

[1] Yang JD, Hainaut P, Gores GJ (2019). A global view of hepatocellular carcinoma: trends, risk, prevention and management. Nature reviews Gastroenterology & hepatology.

[2] Sapisochin G, Bruix J (2017). Liver transplantation for hepatocellular carcinoma: outcomes and novel surgical approaches. Nature reviews Gastroenterology & hepatology.

[3] Bhoori S, Mazzaferro V (2014). Current challenges in liver transplantation for hepatocellular carcinoma. Best practice & research Clinical gastroenterology.

[4] Gao T, Xia Q, Qiu DK (2013). Comparison of survival and tumor recurrence rates in patients undergoing liver transplantation for hepatitis B-related hepatocellular carcinoma using Milan, Shanghai Fudan and Hangzhou criteria. Journal of digestive diseases.

[5] Verna EC, Patel YA, Aggarwal A (2020). Liver transplantation for hepatocellular carcinoma: Management after the transplant. American journal of transplantation: official journal of the American Society of Transplantation and the American Society of Transplant Surgeons.

[6] Özdemir F, Baskiran A (2020). The Importance of AFP in Liver Transplantation for HCC. Journal of gastrointestinal cancer.

[7] Hoffman D, Mehta N (2021). Recurrence of hepatocellular carcinoma following liver transplantation. Expert review of gastroenterology & hepatology.

[8] von Felden J, Garcia-Lezana T, Schulze K (2020). Liquid biopsy in the clinical management of hepatocellular carcinoma. Gut.

[9] Zhou J, Yu L, Gao X (2011). Plasma microRNA panel to diagnose hepatitis B virus-related hepatocellular carcinoma. Journal of clinical oncology: official journal of the American Society of Clinical Oncology.

[10] Fan J, Zhou J, Xu Y (2006). [Indication of liver transplantation for hepatocellular carcinoma: Shanghai Fudan Criteria]. Zhonghua yi xue za zhi.

[11] Hu J, Wang Z, Liao BY (2014). Human miR-1228 as a stable endogenous control for the quantification of circulating microRNAs in cancer patients. International journal of cancer.

[12] Ju MR, Yopp AC (2020). Evolving thresholds for liver transplantation in hepatocellular carcinoma: A Western experience. Annals of gastroenterological surgery.

[13] Akbulut S, Koc C (2020). Do We Need to Be Limited by Matching Milan Criteria for Survival in Living Donor Liver Transplantation?. Journal of gastrointestinal cancer.

[14] Sarici B, Isik B, Yilmaz S (2020). Management of Recurrent HCC After Liver Transplantation. Journal of gastrointestinal cancer.

[15] Xu J, An P, Winkler CA (2020). Dysregulated microRNAs in Hepatitis B Virus-Related Hepatocellular Carcinoma: Potential as Biomarkers and Therapeutic Targets. Frontiers in oncology.

[16] Farid WR, Verhoeven CJ, de Jonge J (2014). The ins and outs of microRNAs as biomarkers in liver disease and transplantation. Transplant international: official journal of the European Society for Organ Transplantation.

[17] Han ZB, Zhong L, Teng MJ (2012). Identification of recurrence-related microRNAs in hepatocellular carcinoma following liver transplantation. Molecular oncology.

[18] Xie QY, Almudevar A, Whitney-Miller CL (2016). A microRNA biomarker of hepatocellular carcinoma recurrence following liver transplantation accounting for within-patient heterogeneity. BMC medical genomics.

[19] Morita K, Shirabe K, Taketomi A (2016). Relevance of microRNA-18a and microRNA-199a-5p to hepatocellular carcinoma recurrence after living donor liver transplantation. Liver transplantation: official publication of the American Association for the Study of Liver Diseases and the International Liver Transplantation Society.

[20] Wang PX, Xu Y, Sun YF (2021). Detection of circulating tumour cells enables early recurrence prediction in hepatocellular carcinoma patients undergoing liver transplantation. Liver international: official journal of the International Association for the Study of the Liver.

[21] Sugimachi K, Matsumura T, Hirata H (2015). Identification of a bona fide microRNA biomarker in serum exosomes that predicts hepatocellular carcinoma recurrence after liver transplantation. British journal of cancer.

[22] Han ZB, Chen HY, Fan JW (2012). Up-regulation of microRNA-155 promotes cancer cell invasion and predicts poor survival of hepatocellular carcinoma following liver transplantation. Journal of cancer research and clinical oncology.

[23] Hamdorf M, Kawakita S, Everly M (2017). The Potential of MicroRNAs as Novel Biomarkers for Transplant Rejection. Journal of immunology research.

[24] Shaked A, Chang BL, Barnes MR (2017). An ectopically expressed serum miRNA signature is prognostic, diagnostic, and biologically related to liver allograft rejection. Hepatology (Baltimore, Md).

[25] Ng KT, Lo CM, Wong N (2016). Early-phase circulating miRNAs predict tumor recurrence and survival of hepatocellular carcinoma patients after liver transplantation. Oncotarget.

[26] Farid WR, Pan Q, van der Meer AJ (2012). Hepatocyte-derived microRNAs as serum biomarkers of hepatic injury and rejection after liver transplantation. Liver transplantation: official publication of the American Association for the Study of Liver Diseases and the International Liver Transplantation Society.

[27] Hu J, Wang Z, Tan CJ (2013). Plasma microRNA, a potential biomarker for acute rejection after liver transplantation. Transplantation.

